# Novel oil spill indices for sentinel-2 imagery: A case study of natural seepage in Qaruh Island, Kuwait

**DOI:** 10.1016/j.mex.2023.102520

**Published:** 2023-12-12

**Authors:** Mohamed Zakzouk, Islam Abou El-Magd, Elham M Ali, Abdulaziz M Abdulaziz, Amjad Rehman, Tanzila Saba

**Affiliations:** a Environment Division, National Authority for Remote Sensing and Space Sciences, Cairo, Egypt; b Mining, Petroleum, & Metallurgical Engineering Department, Faculty of Engineering, Cairo University, Giza, Egypt; c Artificial Intelligence & Data Analytics Lab, College of Computer and Information Sciences, Prince Sultan University, Riyadh, Saudi Arabia

**Keywords:** Oil spill detection, Qaruh island, Sentinel-2 imagery, Spectral analysis, Jeffries-matusita distance, Novel Oil Spill Indeices for Sentinel-2 Imagery

## Abstract

Oil spills are a paramount and immediate challenge affecting marine ecosystems globally. Effective and timely monitoring tools, such as oil detection indices, offer a swift means to track oil spill spread across vast oceanic expanses. Moreover, these indices enhance data clarity, making it more conducive for machine learning and deep learning algorithms.

This study leverages the natural seepage occurring around Qaruh Island, Kuwait as a unique context for the spectral analysis of oil spills using Sentinel-2 multispectral imagery due to repeated occurrences in the same region. This research evaluated 859 single band and 455 multichannel combinations to identify the most effective combinations in oil-water separability, employing the Jeffries-Matusita (JM) distance measure as a key metric. Bands 1, 2, 3, 8A, 11, and 12 consistently featured among the top-performing indices combinations $\frac{B1-B11}{B1+B11};\frac{B1+B2}{B3+B11};\frac{B1+B2}{B3+B12};\frac{B1+B2}{B3+B8A}$ affirming the significant effect of oil spills on visible, Near-Infrared (NIR), and Shortwave Infrared (SWIR) bands. Notably, the indices developed in this study outperformed those from prior research in terms of suitability to unsupervised classification algorithms. A significant conclusion of this study is that incorporating a higher number of bands in the analysis did not correlate with an increase in JM values, suggesting that the selection of specific, informative bands is more critical than the volume of input data. These findings underscore the indispensable role of Sentinel-2 imagery in environmental investigations and highlight the potential for focused, efficient analysis using strategic band combinations for effective oil spill detection.

• This study identified optimized Sentinel-2 band combinations for oil-water separability, benefiting from naturally occurring spills around Qaruh Island.
• The proposed indices outperformed the previous indices for oil spill visualization and clustering.
• The new indices highlighted the critical role of specific band selection over the volume of input data for effective oil spill detection.

## Graphical abstract

**Figure:**
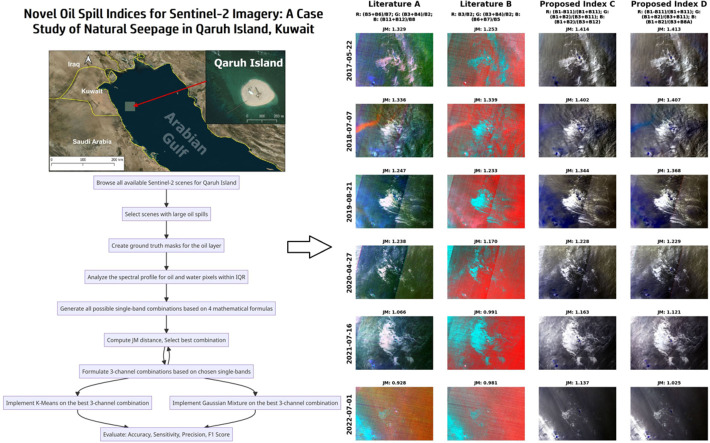

Specifications table

**Table:** 

Subject area:	Environmental Science
More specific subject area:	Oil Spill Detection
Name of your method:	Novel Oil Spill Indeices for Sentinel-2 Imagery
Name and reference of original method:	S. Rajendran, P. Vethamony, F.N. Sadooni, H.A.-S. Al-Kuwari, J.A. Al-Khayat, H. Govil, S. Nasir, Sentinel-2 image transformation methods for mapping oil spill-A case study with Wakashio oil spill in the Indian Ocean, off Mauritius, MethodsX. 8, (2021), 101,327. 10.1016/j.mex.2021.101327
Resource availability:	Data: - Copernicus Open Access Hub: https://scihub.copernicus.eu/dhus/ - Google Earth Engine https://earthengine.google.com/ - EO Browser: https://apps.sentinel-hub.com/eo-browser/ Software: - Q-GIS: https://www.qgis.org/en/site/forusers/download.html Python Packages: - File handling and utilties: os, shutil, csv - Raster data processing: rasterio, rasterio.warp - Data manipulation: pandas, numpy, scipy.stats, scipy.linalg. - Visualization: matplotlib.pyplot, seaborn matplotlib.colors - Machine Learning: sklearn.preprocessing, sklearn.cluster, sklearn.mixture, sklearn.metrics. - Miscellaneous Utilities: itertools, re, textwrap, datetime

## Introduction

Marine oil spills represent a significant environmental challenge with far-reaching ecological and economic consequences. Over the years, a myriad of remote sensing technologies like multispectral [1,2], hyperspectral [3], and Synthetic Aperture Radar (SAR) [4], [5], [6] sensors have been developed to detect and monitor these spills, ensuring timely intervention and mitigation. Multispectral and hyperspectral remote sensing analyze data across multiple spectral bands to differentiate oil slicks from surrounding surfaces while SAR sends and receives reflected waves in microwave range carrying information about surface physical properties.

SAR stands out as a particularly advanced tool among other technologies, offering superior detection capabilities compared to traditional multispectral methods [4,7,8]. This is largely due to SAR's ability to penetrate cloud cover and operate effectively at night, providing detailed imagery of the ocean's surface.

However, SAR and Optical sensors offers high spatial resolution, discontinuity in data due to satellite revisit time can sometimes limit its use. Also, there is significant variability in oil spills’ manifestation in SAR images depending on the type of oil, weather conditions, and sea state [9,10]. To address this, researchers and environmentalists often combine SAR data with optical data [11]. This fusion not only enhances the temporal coverage but also provides a richer, more detailed view of the affected areas. A deep understanding of the spectral features of oil spills is crucial in this context. By recognizing these unique spectral signatures, visualization techniques can be significantly enhanced, allowing for clearer differentiation between oil-contaminated and uncontaminated waters and facilitating more effective response strategies. In change detection studies, multispectral data is employed to analyze and identify alterations in the landscape or environment over time. It enables to detect and understand changes in land cover, vegetation, or other features by comparing different spectral signatures recorded in multiple bands of the spectrum [12], [13], [14].

Oil spills, when they occur in aquatic environments, exhibit distinct spectral characteristics that set them apart from the surrounding clean waters. These spectral features are primarily influenced by the physical and chemical properties of the oil, as well as its interaction with sunlight and the underlying water column. In the visible spectrum, oil spills often appear darker than the surrounding waters due to their reduced reflectance. This is a result of the oil's ability to absorb more sunlight and scatter less light back to the sensor. In the infrared spectrum, the difference becomes even more pronounced. Oil has a unique absorption feature, especially in specific infrared wavelengths [15], which can be used to distinguish it from other natural sea surface films or phenomena. Additionally, the thickness and type of oil, whether it's a light sheen or a thicker crude, can further influence its spectral signature [16].

Remote sensing indices are quantitative measures based on mathematical calculations between multi-bands designed to assess and characterize the severity and extent of certain parameter from optical remote sensing data. By analyzing specific spectral or spatial patterns associated with oil spills, these indices provide valuable information for monitoring and evaluating the impact of oil contamination on the environment. The development of accurate and reliable oil spill indices is crucial for effective oil spill management and mitigation strategies.

Indices provide rapid solution to detect and monitor the spread of oil spill incidents in the vast expanses of oceans [17,18]. These mathematical combinations of different spectral bands simplify the complexity of raw satellite data, making it more accessible and understandable [19,20]. By simplifying these data, indices ensure a clear delineation of oil-contaminated areas, reducing the false positives that can result from atmospheric interferences or water column dynamics. Not only do indices offer a rapid means to extract relevant information from vast datasets, but they also enhance data clarity by reducing noise and highlighting specific features of interest. The distilled data from these indices provides not just a swift snapshot of oil but also a clear dataset ready for machine learning and deep learning algorithms [21,22].

Most developed remote sensing indices are based on the ratio or difference between two bands, like the Normalized Difference Vegetation Index (NDVI) [23,24], but no rule says it has to be this way. A more complex formula involving multiple bands works best. Different spectral band ratios and indices have been used to detect oil spills, as shown in Table 1. These include:

• Vegetation Indices (VIs): Oil spills cause some biophysical and biochemical changes of the vegetation, leading to changes in their spectral signature detected by vegetation indices. Five spectral indices NDVI= (NIR – R)/(NIR + *R*), soil-adjusted vegetation index SAVI= (NIR – R)/(NIR + *R* + 0.5)) × (1 + 0.5) [25], adjusted resistant vegetation index ARVI2= (−0.18 + 1.17 × (NIR – R)/(NIR + *R*)) [26], green near-infrared GNIR= (G – R)/(G + R) [27] and green shortwave infrared GSWIR = *G*/SWIR [28] consistently exhibited sensitivity to the effects of oil pollution [29].
• Normalized Difference Water Index (NDWI): NDWI can help to identify water bodies, making it easier to differentiate between water and oil. It is commonly used to enhance the feature of open water in satellite imagery. The formula for NDWI is NDWI = (Green - NIR) / (Green + NIR) [30,31].
• Modified Normalized Difference Water Index (MNDWI): This index is similar to NDWI but offers improved differentiation between water and other elements. It uses the green and mid-infrared bands to enhance the open water features while suppressing built-up and vegetation signals. the MNDWI formula is: MNDWI = (Green – SWIR1) / (Green + SWIR1) [32,33]. The green band is useful for showing water bodies, and the shortwave infrared band can show features like cloud penetration and the differentiation of snow and ice. By using these two bands, the MNDWI can effectively differentiate water bodies, even in the presence of elements such as ice and snow.
• New Water Index (NWI) was developed, similar to previously developed water indices, based on the Blue and SWIR2 bands [34]. Formula is: NWI = (Blue – SWIR2) / (Blue + SWIR2).
• Sentinel Water Mask (SWM) [35]: New index for detecting water bodies on Sentinel-2 images and gives values ranging from 0 to 2 to best separate water bodies.
• Normalized Burn Ratio (NBR) [36]: While originally used to identify burn areas in vegetation, it has also been used in oil spill detection due to the spectral behavior similarities of burned vegetation and oil slicks in the infrared spectrum. It is a commonly used index for identifying burned areas. It's typically calculated using the near-infrared (NIR) and shortwave infrared (SWIR) bands of a satellite image. The NBR formula is: NBR = (NIR - SWIR) / (NIR + SWIR). These bands can be very useful in oil spill detection due to their ability to penetrate the water's surface and interact with submerged or floating oil. The NBR index is effective for identifying burned areas because healthy vegetation reflects NIR light strongly and absorbs SWIR light, whereas burned areas reflect less NIR light and more SWIR light. Thus, burned areas will have a lower NBR value than healthy vegetation.
• Shortwave Infrared Index (SWIRI): The SWIRI uses the SWIR band to detect the presence of oil on water surfaces.
• Oil spill index (OSI) [37]: OSI is calculated by summing the bands that represent the shoulders of the absorption bands of oil, which serve as the numerator, and using the band closest to the absorption feature as the denominator. This ratio is used to differentiate oil spills. OSI = (B03 + B04) / B02.

**Table 1:** A comprehensive list of historical indices employed to improve the visualization of oil spills.

Index	Mathematical expression	Sentinel-2 Bands formula
NDVI [23]	(NIR - Red) / (NIR + Red)	(B08 - B04) / (B08 + B04)
SAVI [25]	((NIR – R)/(NIR + *R* + 0.5)) × (1 + 0.5)	((B08 – B04)/(B08 + B04 + 0.5)) × (1 + 0.5)
ARVI2 [26]	(−0.18 + 1.17 × ((NIR – Red)/(NIR + Red)))	(−0.18 + 1.17 × ((B08 – B04)/(B08 + B04)))
G/NIR [27]	(Green – Red)/(Green + Red)	(B03– B04)/(B03+ B04)
G/SWIR [28]	Green/SWIR1	B03/ B11
NDWI [30]	(Green - NIR) / (Green + NIR)	(B03 - B08) / (B03 + B08)
NWI [34]	(Blue – SWIR1)/ (Blue + SWIR2)	(B02 – B12) / (B02 + B12)
MNDWI [32]	(Green – SWIR1) / (Green + SWIR1)	(B03 – B11) / (B03 + B11)
SWM [35]	(Blue + Green)/(NIR + SWIR1)	(B02 + B3) / (B08 + B11)
NBR [36]	(NIR – SWIR1) / (NIR + SWIR1)	(B08 – B11) / (B08 + B11)
OSI [17,38,39]	(Red + Green) / Blue	(B04 + B03) / B02
	R: (B05+ B06)/ B07; G: (B03+ B04)/B02; B: (B011+ B012)/ B08	
	R: B03/ B02; G: (B03+ B04)/B02; B: (B06+ B07)/ B05	

In light of the pressing need for efficient monitoring of marine oil spills, this study aims to enhance current indices. Specifically, this research aims to:

(1) Evaluate the efficacy of existing spectral indices in their ability to distinguish between oil and water, not only in terms of separability but also with respect to enhanced visualization.
(2) Formulating advanced indices that surpass the capabilities of their predecessors, thereby offering more precise detection and richer visualization of oil spills.
(3) Understand the underlying spectral behaviors associated with different oil types and conditions, which would subsequently aid in the development of these improved indices.
(4) Establish a benchmark for future studies, ensuring that subsequent research in this domain is built upon a foundation of rigorous testing and analysis.

These objectives underscore the study's commitment to addressing the global challenge of marine oil pollution through state-of-the-art remote sensing techniques.

## Data and methodology

### Study area

**Fig. 1:**
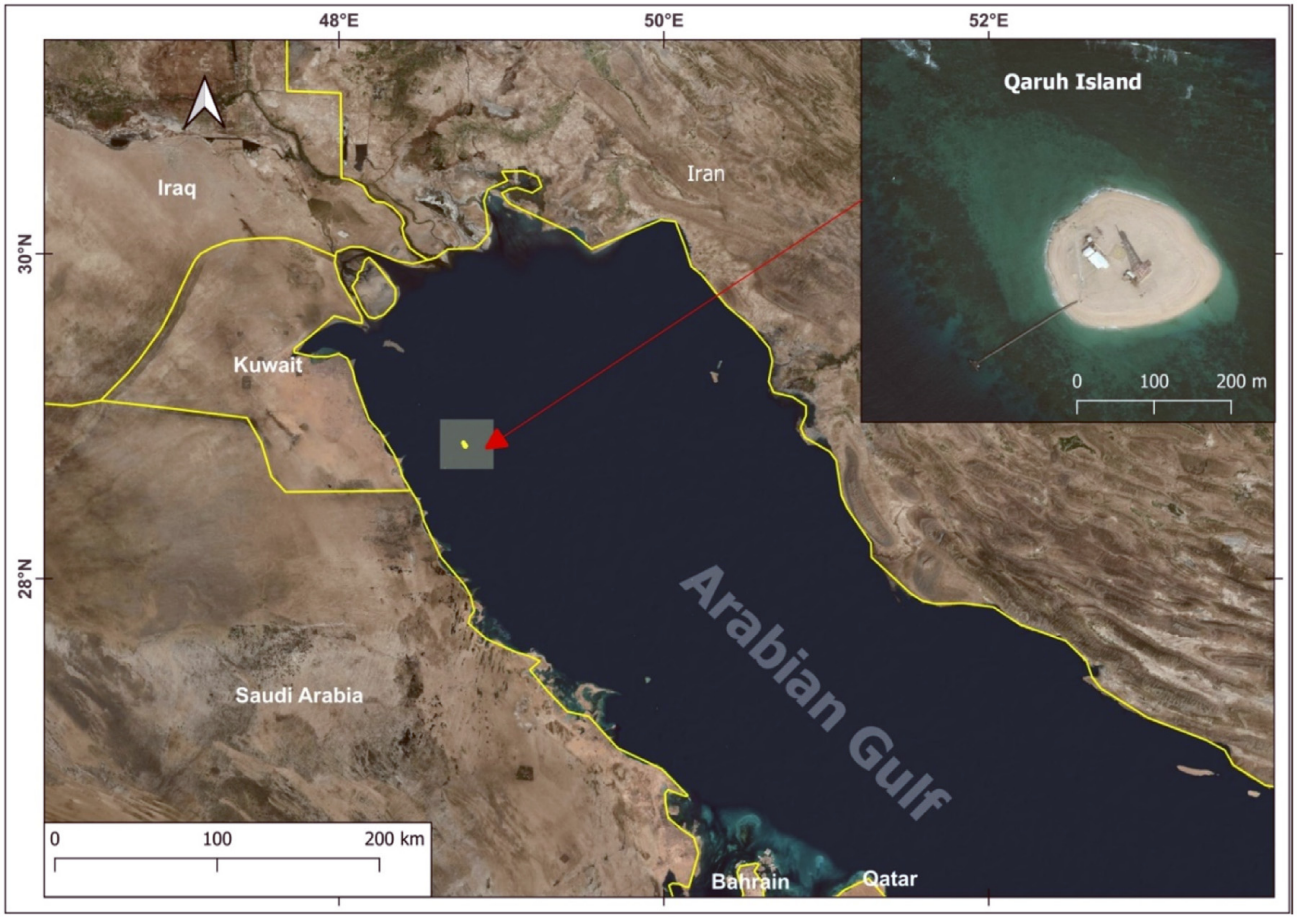
Geographical overview of Qaruh Island.

Qaruh Island, located in the Arabian Gulf as part of the Kuwaiti archipelago, is renowned for its natural seepage phenomenon [40], a rare and intriguing spectacle of petroleum and gas naturally emerging from the sea floor as shown in Fig. 1. This natural occurrence is attributed to the geologic complexities beneath the island, making it one of the few places globally where this spectacle can be witnessed. Essentially, crude oil and gas seep out from underground reservoirs, bubbling up through cracks in the seabed to eventually emerge on the ocean surface. The nature of this seepage results from the intense pressure in the subsurface reservoirs, pushing the hydrocarbons upwards through fractures and fissures.

This location with natural oil seepages provide an unparalleled, recurrent opportunity to understand the dynamics and impacts of oil spills. These seepages, with a history spanning millions of years, are distinct from the abrupt and often devastating anthropogenic oil spills. This consistency could enable to conduct in-depth study, analyzing oil spill spectral and physical characteristics. For the designated study area, a geospatial bounding box was defined with the following coordinates: Northern limit at 48°33′N, Southern limit at 48°58′S, Eastern boundary at 28°57′E, and Western boundary at 28°39′W.

### Data

Sentinel-2 is a multispectral satellite mission under the European Union's Copernicus Programme [41]. It provides 10-m resolution images, covering diverse spectral bands from the visible to the shortwave infrared regions, as illustrated in Table 2. Each of these bands captures distinct wavelengths, providing a rich and detailed representation of the Earth's surface. These capabilities make it particularly suitable for applications like land cover classification, vegetation health assessment, and disaster monitoring, among others.

**Table 2:** Sentinel-2 bands characteristics.

Sentinel 2 Bands	Description	Resolution	Wavelength	
			S2A	S2B
B1	Aerosols	60 m	443.9	442.3
B2	Blue	10 m	496.6	492.1
B3	Green	10 m	560	559
B4	Red	10 m	664.5	665
B5	Red Edge 1	20 m	703.9	703.8
B6	Red Edge 2	20 m	740.2	739.1
B7	Red Edge 3	20 m	782.5	779.7
B8	NIR	10 m	835.1	833
B8A	Red Edge 4	20 m	864.8	864
B9	Water vapor	60 m	945	943.2
B10	Cirrus	60 m	1373.5	1376.9
B11	SWIR 1	20 m	1613.7	1610.4
B12	SWIR 2	20 m	2202.4	2185.7

Google Earth Engine (GEE) offers the Level-1C product specifically for Sentinel-2 imagery. This product which presents the Top of Atmosphere (TOA) reflectance values, has been used for analysis. Band 10 has been omitted from data analysis because it primarily detects high-altitude features like cirrus clouds due to its sensitivity to water vapor absorption and is not suitable for ground-level analysis. All Sentinel-2 images and their fragments presented in this study are copyrighted and used under ESA permission.

### Method details

(i) Data acquisition and selection:

Utilizing the Sentinel Hub Earth Observation browser (website link: https://www.sentinel-hub.com/explore/eobrowser), all available scenes from Landsat 8, Sentinel-1, and Sentinel-2 that covered Qaruh Island between 2017 and 2022 have been visually checked. From this extensive dataset, we specifically selected and downloaded instances of the most significant oil spills via GEE to help better investigate the spectral properties of oil properties in the marine environment.

(ii) Ground truth preparation:

For the chosen Sentinel-2 scenes, oil spill ground truth masks have been established to be used in later results assessment. Masking was accomplished using the Q-GIS software (version 3.22.4), relying on digitization complemented by adaptive thresholding techniques. This method's efficacy is contingent upon the image quality and the discernibility of oil spills from the surrounding waters.

(iii) Spectral profile analysis:

The spectral profiles of oil and water pixels that resided within the data interquartile range (IQR) have been plotted. By emphasizing the IQR, the analysis becomes less susceptible to extreme values, effectively minimizing the impact of potential outliers.

(iv) Band Combination Exploration:

Inspired by the literature, all conceivable band combinations of the mathematical formulations presented in Table 3 have been generated for evaluation. The current study sought to exhaustively test every combination derived from these foundational formulas.

**Table 3:** Proposed mathematical formulas for generating band combinations.

Naming Convention	Band Ratio	Consideration	Available Combinations
Ratio 1	$\frac{Band\;A-Band\;B}{Band\;A+Band\;B}$	Band *A* > Band B	66
Ratio 2	$\frac{Band\;A}{Band\;B}$	Band *A* > Band B	66
Ratio 3	$\frac{Band\;A+Band\;B}{Band\;C}$	Band A ≠ Band B ≠ Band C	220
Ratio 4	$\frac{Band\;A+Band\;B}{Band\;C+Band\;D}$	Band A ≠ Band B ≠ Band C ≠ Band D	495
Generated Combinations			859

(iv) Visualization and contrast assessment of band combinations:

To assess the effectiveness of generated band ratios for better distinguishing between oil spills and surrounding water in Sentinel-2 spectral bands, the Jeffries-Matusita (JM) distance statistical metric has been employed [42]. This measure serves as a robust tool to quantify the separability between two distinct classes within a feature space. Within the context of remote sensing, this measure is often used to determine the best spectral bands or band ratios for distinguishing between two land cover classes [43]. The JM distance operates on a scale ranging from 0 to $\sqrt{2}$ (approximately 1.4142). A value of 0 signifies a complete overlap between the classes, while a score of approximately 1.4142 denotes absolute separability.

The JM distance between two classes A and B is calculated as in Eq. (1):

(1) $$JM=\sqrt{2\left(1-e^{-B}\right)}$$

Where the Bhattacharyya distance for two classes is given by Eq. (2):

(2) $$B=\frac{1}{8}{\left(\mu_{1}-\mu_{2}\right)}^{T}\sum {\left(\mu_{1}-\mu_{2}\right)}^{-1}+\frac{1}{2}\ln\left(\frac{det\left(\Sigma \right)}{\det\left(\Sigma_{1}\right)\times det\left(\Sigma_{2}\right)}\right)$$

Where: $\mu_{1},\;\mu_{2}$ are the mean vectors of the two classes; Σis the average covariance matrix

$\Sigma_{1},\;\Sigma_{2}$ are covariance matrices of the two classes.

Also, visual inspection and interpretation as one of the simplest ways to judge the best band ratio based on contrast between the oil spill and the surrounding water has been combined in analysis.

(v) Selection and assessment of optimal band combinations:

Upon computing JM distances, the most effective band combination have been identified. This optimal combination was then integrated into three other channel combinations. Subsequently, the JM distances for these multiband images have been recalculated, ensuring a comprehensive evaluation to ultimately pinpoint the premier multiband combination.

(vi) Unsupervised clustering for accuracy assessment:

For the distinguished multiband combination, two-class unsupervised clustering approaches have been used. These served as an additional metric to gauge the accuracy of developed indices in comparison to previously established ones.

Data classification without prior knowledge of the categories relies on inherent data properties, and group similar data pixels. The K-means and the Gaussian Mixture Model Algorithms were employed in this regard. The strength of Two-Class unsupervised clustering lies in its capacity to rapidly discern contrasting features within a dataset, making it particularly useful selecting best band combinations.

(a) K-means clustering

It aims to partition n_samples into n_clusters in which each sample belongs to the cluster whose mean is closest to it [44]. The objective function that K-means minimizes is the within-cluster sum of squares as given by Eq. (3):

(3) $$J=\sum_{i=1}^{k}\Sigma_{\mu_{j}\in C_{i}}\left(∥x_{j}-\mu_{i}{∥}^{2}\right)$$

Where: J represents the total within-cluster variance; K is the number of desired clusters; C_i_ denotes the i^th^ cluster; $\mu_{j}\;$is the i^th^ cluster center; $x_{i}$ is a data point belonging to cluster C_i_; ∥.∥ is the Euclidean distance

(b) Gaussian Mixture Model (GMM):

GMM is a probabilistic model that assumes that the data is generated from a mixture of several Gaussian distributions [45]. Each of these Gaussian distributions represents a cluster. Hence, a GMM tries to fit these Gaussian distributions to the data.

Mathematical Principle:

Given a dataset, the objective is to maximize the likelihood of the parameters of the model, given the data. If we have K Gaussian components, the likelihood for the entire dataset $X=\{x_{1},x_{2},\ldots .x_{N}\}$ is given by Eqs. (4) and (5):

(4) $$L\left(\theta |X\right)=\sum_{i=1}^{K}\pi_{k}N\left(x_{i}|\mu_{k},\sum k\right)$$

(5) $$N\left(x_{i}|\mu_{k},\sum k\right)=\frac{1}{{\left(2\pi \right)}^{d/2}{\left|\Sigma \right|}^{1/2}}exp\left(-\frac{1}{2}{\left(x-\mu \right)}^{T}\Sigma^{-1}\left(x-\mu \right)\right)$$

Where: x is the data point; k is the number of Gaussian distributions; $\pi_{k}$ are the mixture coefficients that represent the probability that a randomly selected x originated from component k.

$N(x_{i}|\mu_{k},\sum k)$ is the Gaussian distribution function with mean $\mu_{k}$and covariance ∑k and d is the dimensionality of the data

θ represent represents the parameters of the model (i.e., the means $\mu_{k}$, covariances ∑k, and mixture coefficients $\mu_{k}$ of each Gaussian component).

It models data as being generated from a mixture of several Gaussian distributions, and then assigns each data point to one of these distributions.

(c) Accuracy Assessment

Accuracy assessment of clustered classes has been done to ensure the reliability and credibility of proposed indices. Key metrics used in this assessment include accuracy, sensitivity, precision and F1 scores. These metrics provide insights into the proportion of correctly classified pixels, the likelihood of misclassification, and the agreement between the mask and the reference data beyond random chance as follow:

True Positives (tp): The number of positive instances (labeled as 1) that were correctly classified as positive by the model. $t_{p}=\sum \left(\text{mask}=1\;\wedge \text{truth}=1\right)$

False Negatives (fn): The number of positive instances that were incorrectly classified as negative (0) by the model. $f_{n}=\sum \left(\text{mask}=0\;\wedge \text{truth}=1\right)$

False Positives (fp): The number of negative instances (labeled as 0) that were incorrectly classified as positive by the model. $f_{p}=\sum \left(\text{mask}=1\;\wedge \text{truth}=1\right)$

True Negatives (tn): The number of negative instances that were correctly classified as negative by the model. $t_{n}=\sum \left(\text{mask}=0\;\wedge \text{truth}=0\right)$

Sensitivity: The proportion of actual positive instances that were correctly classified. It shows how well the model can detect positive instances. $Sensitivity=\frac{t_{p}}{t_{p}+f_{n}}$

Precision: The proportion of positive identifications by the model that were actually correct. It shows how many of the instances that the model labeled as positive are indeed positive. $Precision=\frac{t_{p}}{t_{p}+f_{p}}$

F1 Score: The harmonic mean of precision and sensitivity. It is a balance between precision and sensitivity. $F1\;=\frac{2\;\times t_{p}}{2\;\times t_{p}+f_{p}+f_{n}}$

Accuracy: The proportion of all instances that were correctly classified, both positive and negative. $\text{Accuracy}=\frac{t_{p}+t_{n}}{t_{p}+t_{n}+f_{p}+f_{n}}$.

**Fig. 2:**
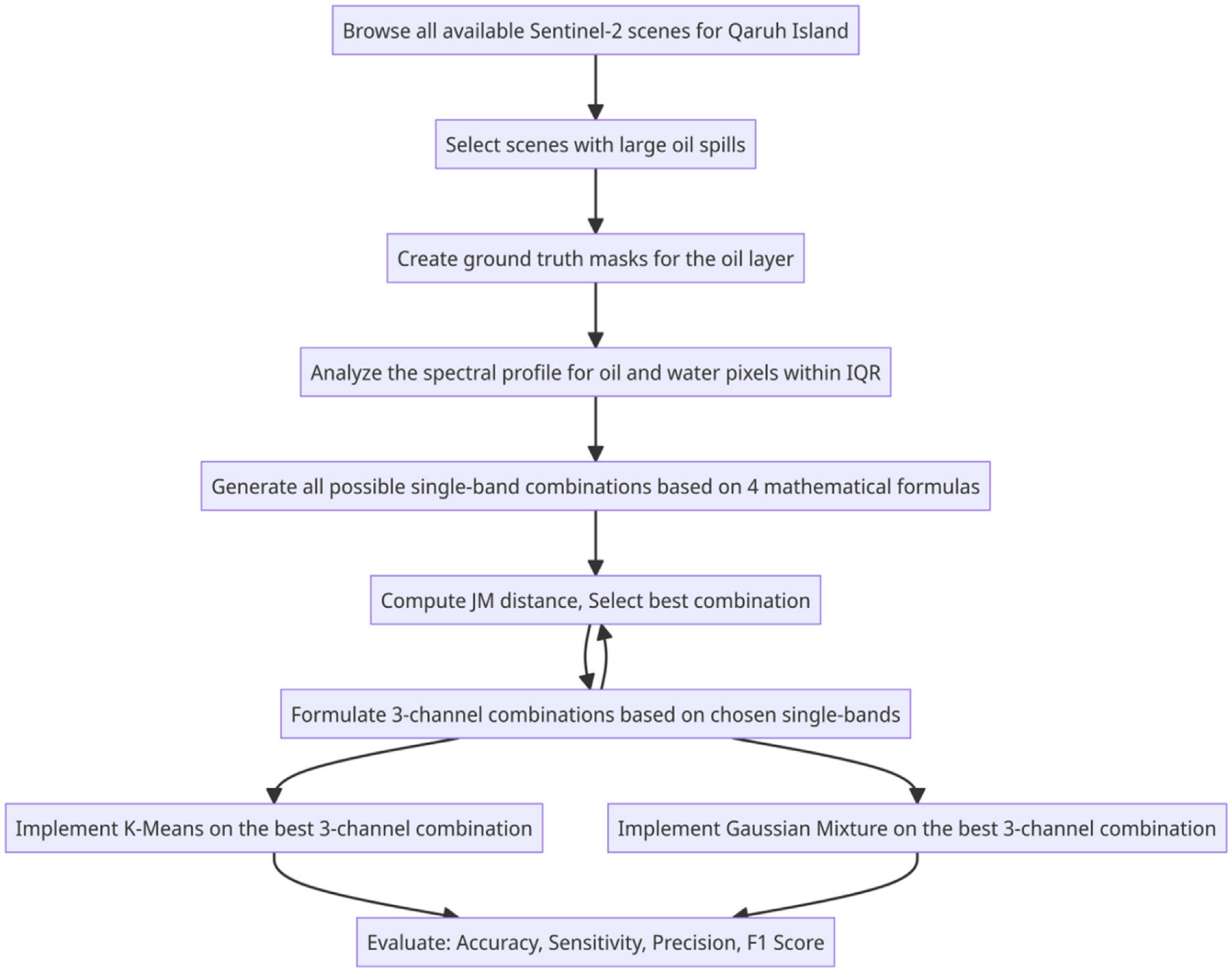
Flowchart depicting the algorithm for developing and assessing the best oil spill index.

A simplified illustration of the sequence of analysis is illustrated in Fig. 2.

In the course of analysis, several Python libraries have been employed to facilitate data processing, visualization, and statistical analysis. The following provides a comprehensive list of these libraries:

- Basic utilities and file handling: os, shutil, and csv for general file operations and directory management.
- Raster data processing: The rasterio library was instrumental in handling raster datasets, while rasterio.warp was used for reprojecting and resampling raster data.
- Data manipulation and analysis: pandas and numpy, and statistical functions were derived from scipy.stats and scipy.linalg.
- Visualization: Graphical representations were crafted using matplotlib.pyplot, with seaborn enhancing the visual aesthetics. The matplotlib.colors module provided additional color normalization functionalities.
- Machine Learning and Data Preprocessing: The sklearn library was extensively used, with modules such as sklearn.preprocessing for data scaling, sklearn.cluster for clustering algorithms like KMeans, and sklearn.mixture for Gaussian Mixture models. Model evaluation metrics like accuracy, recall, precision, and F1 score were sourced from sklearn.metrics.
- Miscellaneous Utilities: itertools for creating combinations, re for regular expression operations, textwrap for text formatting, and datetime for handling date and time operations.

## Results and validation

**Fig. 3:**
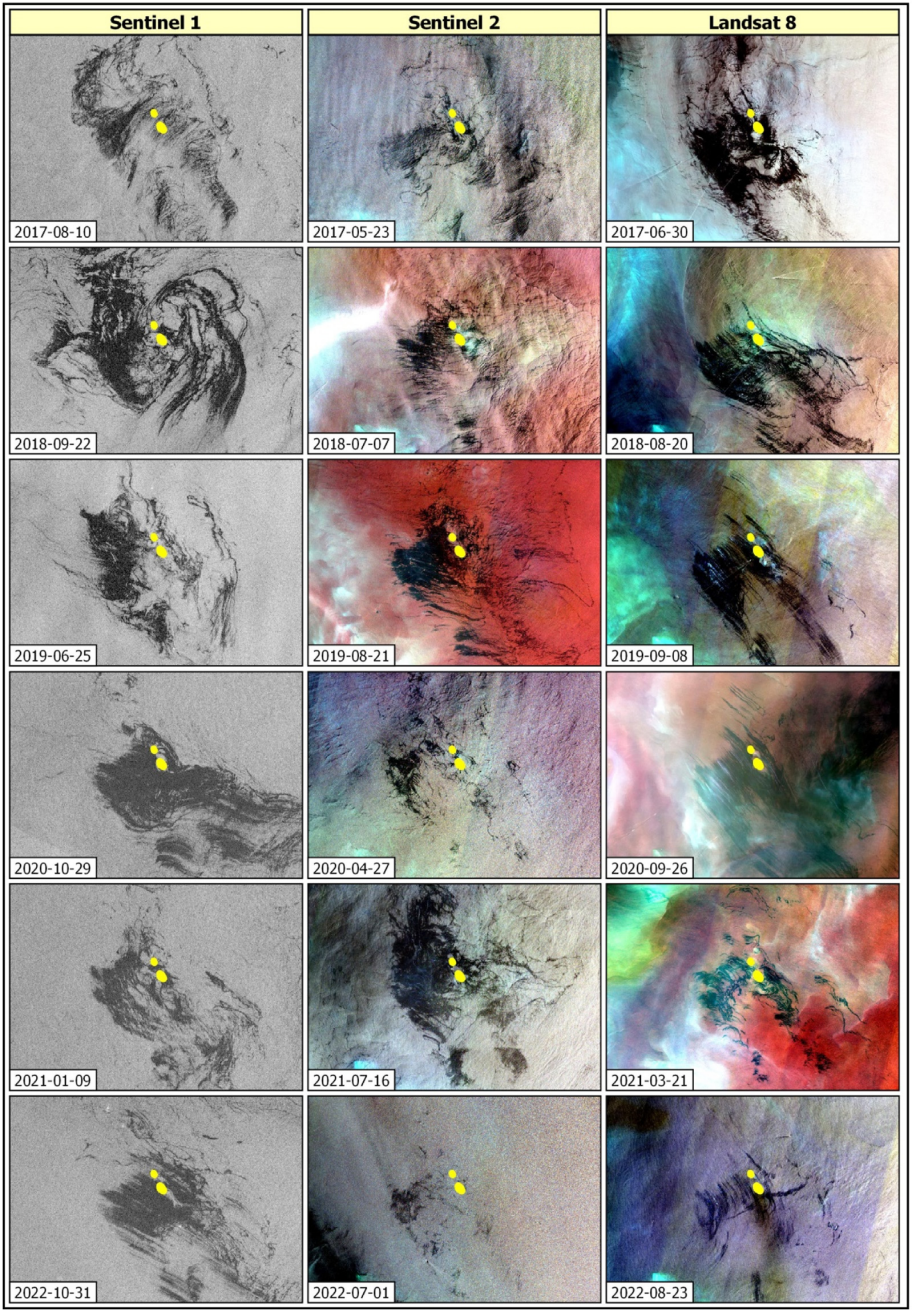
Illustrative depictions of large oil spill incidents surrounding Qaruh Island as captured in Sentinel-1, Sentinel-2, and Landsat 8 imagery.

During the course of the study, a substantial number of satellite scenes encompassing the area of interest: 648 from Sentinel-1, 323 from Sentinel-2, and 242 from Landsat were explored. From this extensive collection, six scenes from each satellite mission that prominently featured large oil spill incidents were selected. These chosen scenes serve as the foundation for cross-analysis, as depicted in Fig. 3.

**Fig. 4:**
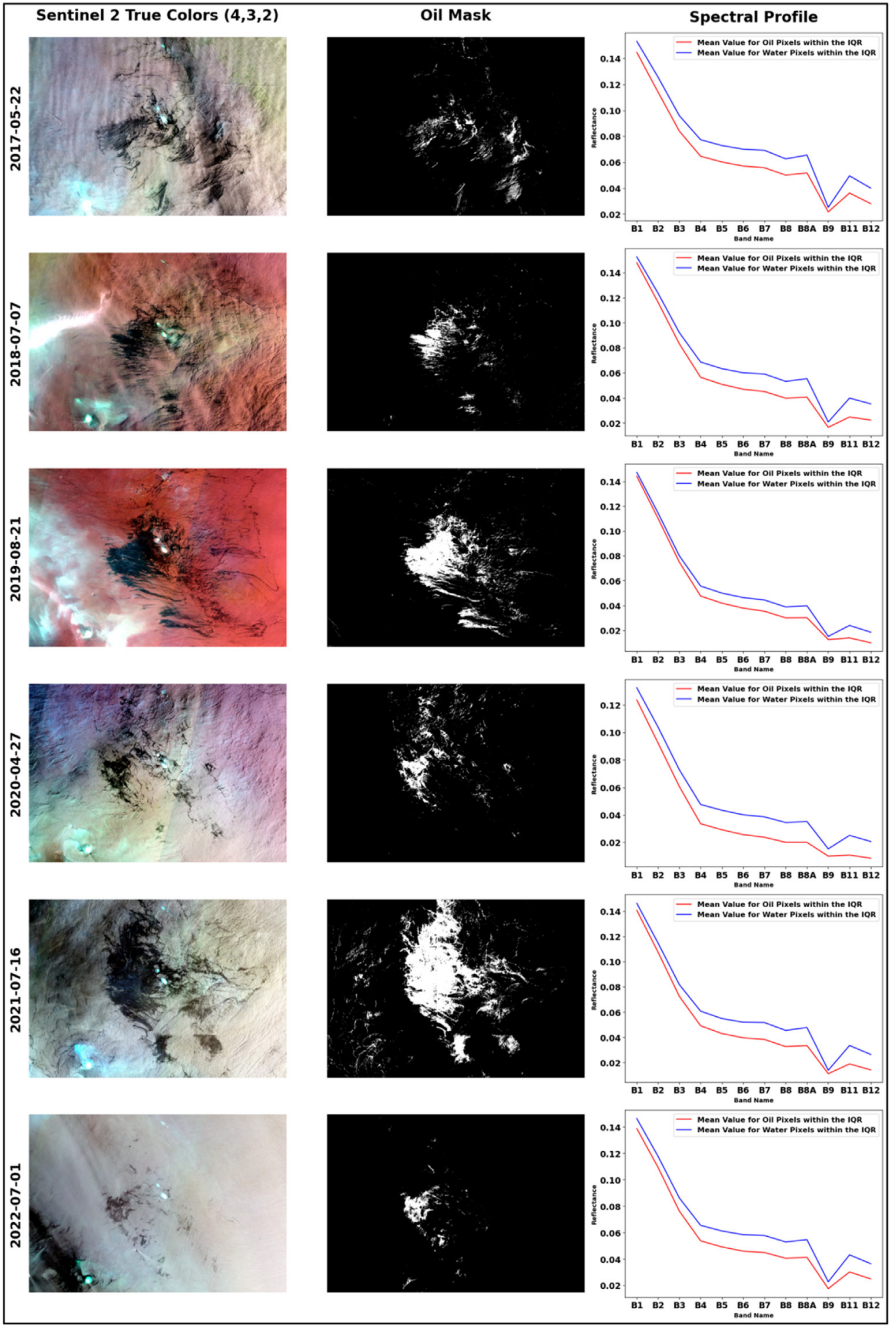
Spectral signature analysis: reflectance patterns within the interquartile data range (IQR).

In the spectral profile analysis of oil pixels, specifically those within the interquartile range (IQR) from Sentinel-2 imagery, oil spill data revealed a distinct pattern, as shown in Fig. 4. The reflectance profile for oil was markedly lower than that of water, especially in the bands B8, B8A, B11, and B12. This observation aligns with the fact that darker oil typically results in lower reflectance values.

Table 4 presents the calculated decrease ratio between water and oil means. Notably, bands 11 and 12 exhibit a significant decline, with the spectral profile for oil showing approximately a 40 % reduction in comparison to water.

**Table 4:** Spectral profile decrease ratio between water and oil means.

	Decrease Ratio Between Water and Oil Means (%)						
Band	2017–05–22	2018–07–07	2019–08–21	2020–04–27	2021–07–16	2022–07–01	Average
B1	5.54	3.06	2.14	6.68	3.80	5.16	4.39
B2	9.19	5.86	3.77	11.15	6.27	7.12	7.23
B3	12.40	9.69	6.35	16.75	11.35	11.52	11.34
B4	16.51	17.77	14.51	29.38	19.11	17.91	19.20
B5	17.40	19.77	16.25	32.69	21.64	19.68	21.24
B6	18.56	21.90	18.37	35.95	23.77	21.31	23.31
B7	19.43	23.68	20.39	38.38	25.93	22.33	25.03
B8	20.01	25.21	22.69	41.84	28.24	23.31	26.88
B8A	20.98	26.71	24.23	43.00	29.94	24.45	28.22
B9	14.00	20.43	17.02	34.78	19.46	22.67	21.39
B11	26.77	37.85	42.06	57.00	43.63	30.00	39.55
B12	30.00	36.77	46.22	58.74	46.36	31.53	41.60

Table 5 presents the JM distance calculations for the 859 formulated combinations, evaluated across data ranges of 50, 80, 90, and 100 %. The table highlights the top five combinations for each band ratio. A comprehensive assessment reveals that Ratio 1 and Ratio 4 exhibit superior separability. Conversely, Ratio 3 lags behind in distinguishing capabilities. Interestingly, individual bands demonstrated enhanced separability compared to Ratio 2.

**Table 5:** Leading bands ranked by JM distance across diverse data ranges.

Data Range	JM distance				
	One Band	Ratio 1	Ratio 2	Ratio 3	Ratio 4
(0–100)%	B12:1.129 B11:1.098 B6:1.095 B5:1.095 B8A:1.092	(B1, B12):1.163 (B1, B11):1.153 (B2, B12):1.150 (B2, B11):1.137 (B3, B12):1.128	(B1, B12):1.110 (B1, B8A):1.106 (B1, B11):1.106 (B1, B7):1.104 (B1, B5):1.103	(B1, B9, B12):1.109 (B1, B8A, B12):1.109 (B1, B5, B12):1.108 (B1, B6, B12):1.108 (B1, B7, B12):1.108	(B1, B2, B3, B12):1.152 (B1, B2, B3, B11):1.148 (B1, B4, B5, B12):1.133 (B1, B5, B6, B12):1.129 (B1, B2, B4, B12):1.129
(5–95)%	B12:1.284 B11:1.282 B8A:1.275 B6:1.273 B5:1.273	(B1, B12):1.275 (B1, B11):1.271 (B2, B12):1.263 (B2, B11):1.257 (B1, B8A):1.256	(B1, B8A):1.242 (B1, B5):1.241 (B1, B6):1.240 (B1, B7):1.240 (B1, B12):1.240	(B1, B9, B12):1.240 (B1, B9, B11):1.239 (B1, B8A, B12):1.239 (B1, B6, B11):1.239 (B1, B7, B12):1.239	(B1, B2, B3, B12):1.275 (B1, B2, B3, B11):1.273 (B1, B2, B3, B8A):1.261 (B1, B4, B5, B12):1.257 (B1, B2, B3, B7):1.256
(10–90)%	B12:1.337 B11:1.336 B8A:1.332 B5:1.331 B6:1.331	(B1, B12):1.334 (B1, B11):1.332 (B2, B12):1.325 (B1, B8A):1.322 (B2, B11):1.322	(B1, B8A):1.315 (B1, B5):1.315 (B1, B6):1.314 (B1, B7):1.314 (B1, B11):1.309	(B1, B5, B8A):1.309 (B1, B2, B8A):1.309 (B1, B4, B8A):1.309 (B1, B6, B8A):1.309 (B1, B7, B8A):1.309	(B1, B2, B3, B12):1.335 (B1, B2, B3, B11):1.334 (B1, B2, B3, B8A):1.327 (B1, B4, B5, B12):1.325 (B1, B2, B3, B7):1.324
(25–75)%	B11:1.409 B4:1.409 B5:1.409 B6:1.409 B12:1.409	(B1, B11):1.410 (B1, B12):1.410 (B1, B8A):1.409 (B2, B12):1.409 (B2, B11):1.409	(B1, B9):1.409 (B1, B8A):1.409 (B1, B6):1.409 (B1, B5):1.409 (B1, B7):1.409	(B1, B2, B8A):1.408 (B1, B7, B8A):1.408 (B1, B5, B8A):1.408 (B1, B4, B8A):1.408 (B1, B6, B8A):1.408	(B1, B2, B3, B12):1.411 (B1, B2, B3, B11):1.411 (B1, B2, B3, B8A):1.410 (B1, B2, B3, B9):1.410 (B1, B4, B5, B12):1.410
Highest five combinations for all data ranges	B12:1.290 B11:1.281 B6:1.277 B8A:1.277 B5:1.277	(B1, B12):1.295 (B1, B11):1.292 (B2, B12):1.287 (B2, B11):1.281 (B1, B8A):1.278	(B1, B8A):1.268 (B1, B5):1.267 (B1, B7):1.267 (B1, B6):1.267 (B1, B12):1.266	(B1, B9, B12):1.265 (B1, B8A, B12):1.265 (B1, B9, B11):1.265 (B1, B7, B12):1.265 (B1, B6, B12):1.265	(B1, B2, B3, B12):1.293 (B1, B2, B3, B11):1.291 (B1, B2, B3, B8A):1.281 (B1, B4, B5, B12):1.281 (B1, B5, B6, B12):1.279
Overall highest five band combinations	Ratio1(1.287) > Ratio4(1.285)>One Band (1.280)> Ratio2(1.267)>Ratio3(1.265) Ratio 1 (B1, B12) 1.295 - Ratio 4 (B1, B2, B3, B12) - 1.293, Ratio 1 (B1, B11) 1.292 - Ratio 4 (B1, B2, B3, B11) 1.291 - B12 1.290				

**Fig. 5:**
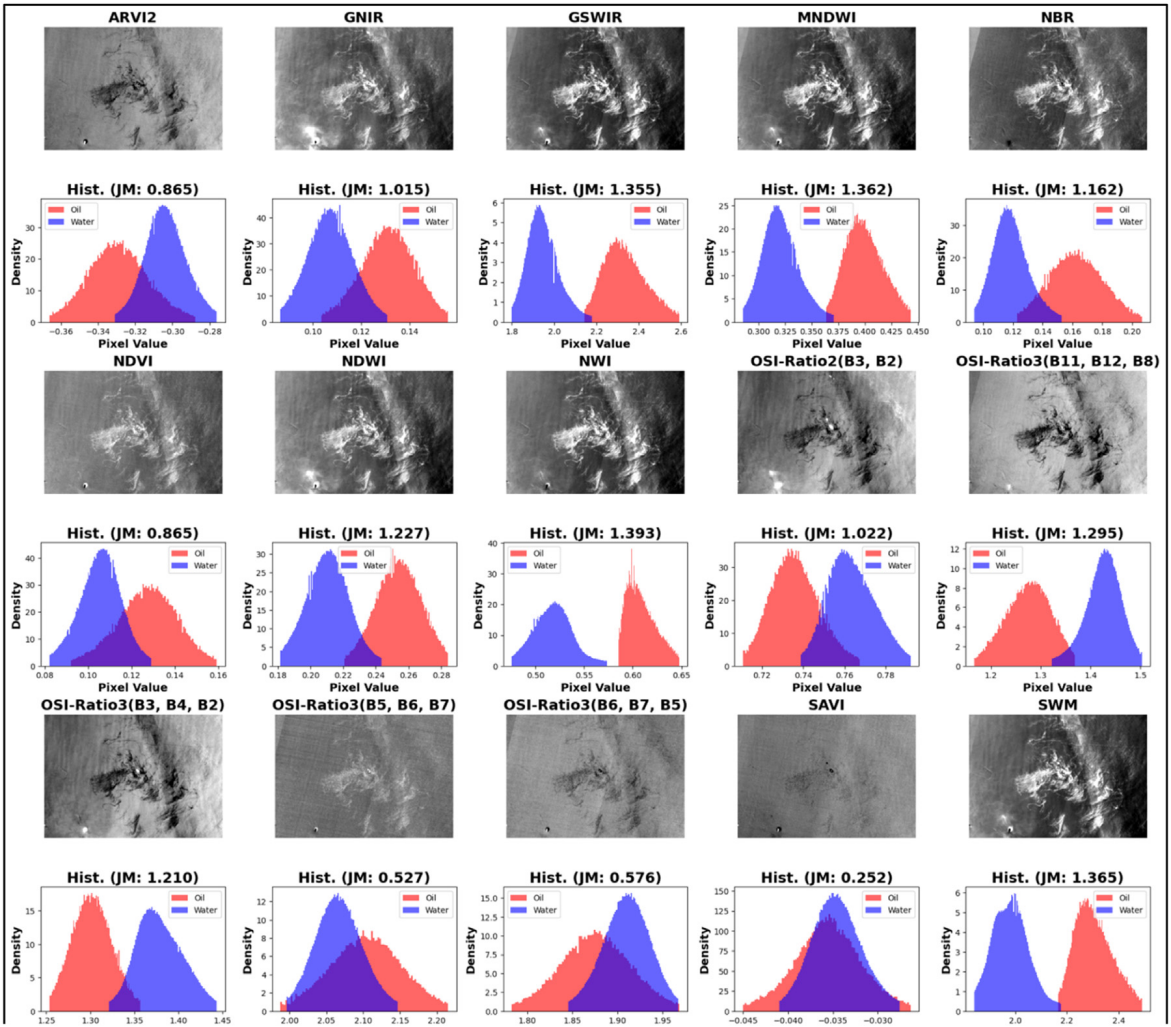
Histograms visualize moderate to low separability of 15 spectral indices derived from literature review.

**Fig. 6:**
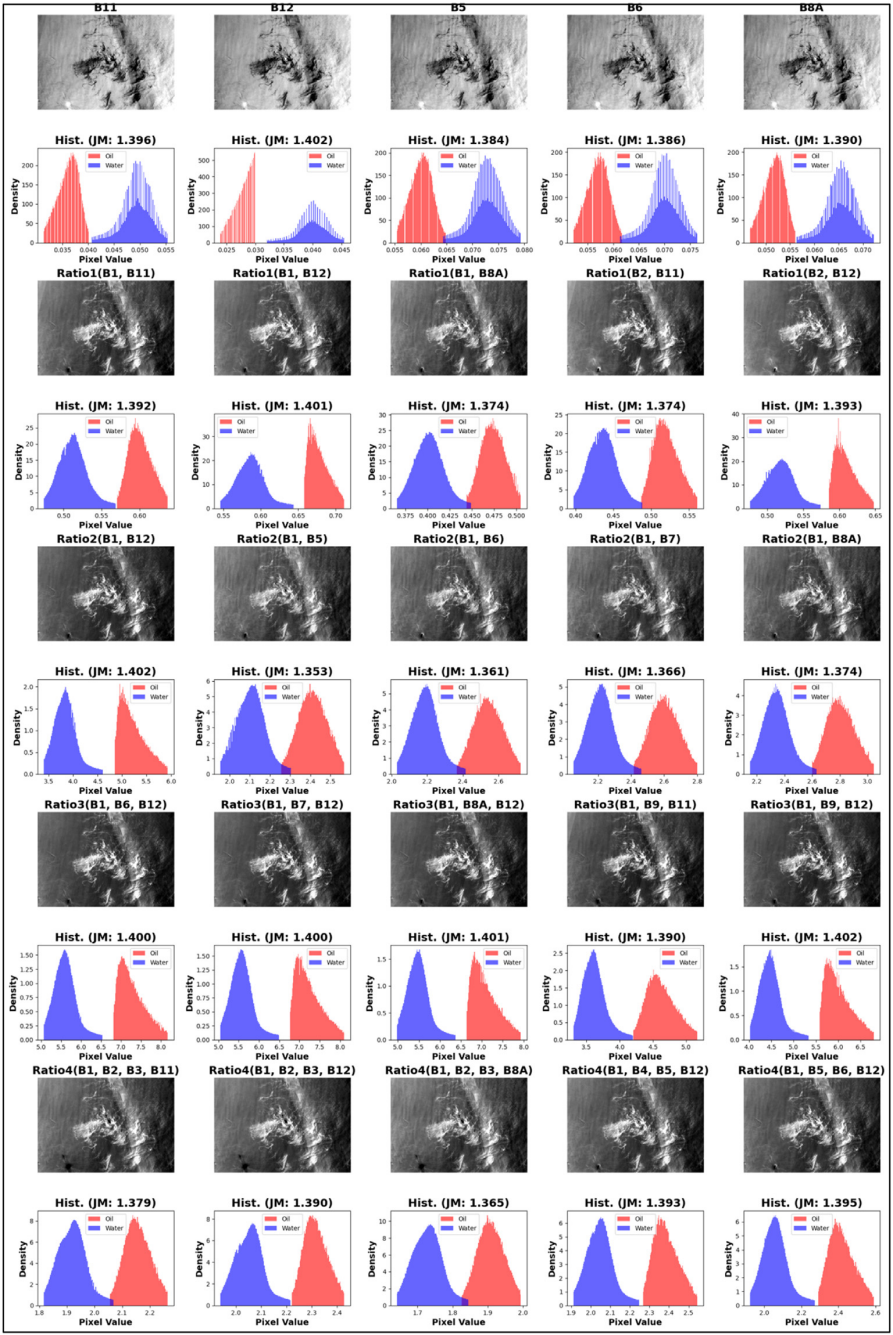
Histograms visualize excellent separability of current study selected spectral indices.

To visually represent the outcomes derived from the JM calculations, histogram plots are employed in Figs. 5, 6. These plots provide a clear graphical representation of the frequency distribution of data points, allowing for an intuitive understanding of the separability between the oil and water layers. By juxtaposing the study-selected band combinations against the established 15 indices derived from literature review, the comparative analysis underscores the efficacy of our selected bands against the well-documented combinations from previous studies. SWM, NWI showed the highest separability among previous studies with JM around 1.36 while all JM of the selected indices in the current study ranged from 1.36 to 1.4.

Drawing from the findings presented, the top 15 band combinations were identified, as detailed in Table 6. Leveraging these selections, three-channel band combinations were formulated, resulting in a total of ​ 455 unique combinations. Subsequent to this, the JM distance calculations were repeated for these three-channel combinations, and the most distinguishable combinations were then shortlisted in Table 7.

**Table 6:** Fifteen selected bands were utilized in the three-channel combinations.

	One Band	Ratio 1	Ratio 4
Highest five combinations for all data ranges	B12:1.290 B11:1.281 B6:1.277 B8A:1.277 B5:1.277	(B1, B12):1.295 (B1, B11):1.292 (B2, B12):1.287 (B2, B11):1.281 (B1, B8A):1.278	(B1, B2, B3, B12):1.293 (B1, B2, B3, B11):1.291 (B1, B2, B3, B8A):1.281 (B1, B4, B5, B12):1.281 (B1, B5, B6, B12):1.279

**Table 7:** Optimal three-channel band combination.

	JM		
Date	Sent 2 12 band image	Fifteen stacked bands	Highest three-channel band combinations
2017–05–22	1.380	1.41408	Ratio1(B1, B11), Ratio4(B1, B2, B3, B11), Ratio4(B1, B2, B3, B12): 1.41411 Ratio1(B2, B11), Ratio4(B1, B2, B3, B11), Ratio4(B1, B2, B3, B12): 1.41389 Ratio4(B1, B2, B3, B11), Ratio4(B1, B2, B3, B12), Ratio4(B1, B5, B6, B12): 1.4127 Ratio1(B1, B11), Ratio4(B1, B2, B3, B11), Ratio4(B1, B2, B3, B8A): 1.41269 Ratio4(B1, B2, B3, B11), Ratio4(B1, B2, B3, B12), Ratio4(B1, B2, B3, B8A): 1.41216
2018–07–07	1.363	1.41417	Ratio1(B1, B8A), Ratio4(B1, B2, B3, B12), Ratio4(B1, B2, B3, B8A): 1.41307 Ratio4(B1, B2, B3, B11), Ratio4(B1, B2, B3, B12), Ratio4(B1, B2, B3, B8A): 1.41164 Ratio1(B1, B12), Ratio4(B1, B2, B3, B12), Ratio4(B1, B2, B3, B8A): 1.41128 Ratio4(B1, B2, B3, B12), Ratio4(B1, B2, B3, B8A), Ratio4(B1, B5, B6, B12): 1.41122 Ratio1(B2, B11), Ratio4(B1, B2, B3, B12), Ratio4(B1, B2, B3, B8A): 1.41116
2019–08–21	1.303	1.41396	Ratio1(B1, B12), Ratio1(B1, B8A), Ratio1(B2, B12): 1.41391 Ratio1(B1, B11), Ratio1(B1, B12), Ratio1(B2, B12): 1.41389 Ratio1(B1, B12), Ratio1(B2, B12), Ratio4(B1, B2, B3, B12): 1.41382 Ratio1(B1, B12), Ratio1(B2, B12), Ratio4(B1, B5, B6, B12): 1.4138 Ratio1(B1, B12), Ratio1(B2, B12), Ratio4(B1, B4, B5, B12): 1.41373
2020–04–27	1.309	1.41258	Band11, Ratio1(B1, B11), Ratio4(B1, B5, B6, B12): 1.35507 Band12, Ratio1(B1, B12), Ratio4(B1, B5, B6, B12): 1.35184 Band11, Ratio1(B1, B11), Ratio4(B1, B4, B5, B12): 1.35117 Band12, Ratio1(B1, B12), Ratio4(B1, B4, B5, B12): 1.34794 Ratio1(B1, B12), Ratio1(B2, B12), Ratio4(B1, B2, B3, B8A): 1.3432
2021–07–16	1.053	1.40711	Band12, Ratio1(B1, B12), Ratio4(B1, B5, B6, B12): 1.27526 Band12, Ratio1(B1, B12), Ratio4(B1, B4, B5, B12): 1.27234 Band11, Ratio1(B1, B11), Ratio4(B1, B5, B6, B12): 1.2679 Band11, Ratio1(B1, B11), Ratio4(B1, B4, B5, B12): 1.26341 Band11, Ratio1(B1, B11), Ratio4(B1, B2, B3, B11): 1.2491
2022–07–01	1.162	1.40400	Ratio1(B1, B8A), Ratio4(B1, B2, B3, B12), Ratio4(B1, B2, B3, B8A): 1.26476 Ratio4(B1, B2, B3, B11), Ratio4(B1, B2, B3, B12), Ratio4(B1, B2, B3, B8A): 1.21689 Ratio1(B1, B11), Ratio4(B1, B2, B3, B12), Ratio4(B1, B2, B3, B8A): 1.20157 Band11, Ratio1(B1, B11), Ratio1(B1, B12): 1.20087 Ratio1(B2, B11), Ratio4(B1, B2, B3, B12), Ratio4(B1, B2, B3, B8A): 1.19444
Overall optimal Combinations			Ratio1(B1, B11), Ratio4(B1, B2, B3, B11), Ratio4(B1, B2, B3, B12):1.414 Ratio1(B2, B11), Ratio4(B1, B2, B3, B11), Ratio4(B1, B2, B3, B12):1.414 Ratio4(B1, B2, B3, B11), Ratio4(B1, B2, B3, B12), Ratio4(B1, B5, B6, B12):1.413 Ratio1(B1, B11), Ratio4(B1, B2, B3, B11), Ratio4(B1, B2, B3, B8A):1.413 Ratio4(B1, B2, B3, B11), Ratio4(B1, B2, B3, B12), Ratio4(B1, B2, B3, B8A):1.412
Most repeated terms in the highest 3-channel combinations		Ratio4(B1, B2, B3, B12): 14; Times Ratio4(B1, B2, B3, B8A): 12 Times Ratio1(B1, B12): 12 Times; Ratio1(B1, B11): 10 Times Ratio4(B1, B2, B3, B11): 8 Times; Ratio4(B1, B5, B6, B12): 7 Times	

**Fig. 7:**
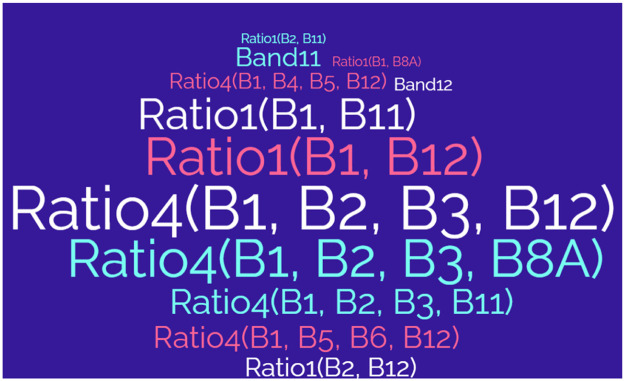
Words map of the most repeated terms in optimal 3-channel band combinations.

In Table 7, the JM distance has been calculated for each original Sentinel-2 12-band image, as well as for the combined set of 15 bands used to create the three-channel combinations. Interestingly, despite the incorporation of a higher number of bands in the analysis, the three-channel combinations were observed to yield higher JM values. This counterintuitive result suggests that, in future studies, it may be possible to streamline the input data for deep learning and machine learning training datasets. Specifically, researchers could potentially focus on utilizing only the most informative components for training, thereby avoiding the need to process all available bands in the image, which can lead to more efficient and focused models. Fig. 7 visualizes the most repeated terms in the optimal three-channel band combinations to guide band selection for future research endeavors.

**Fig. 8:**
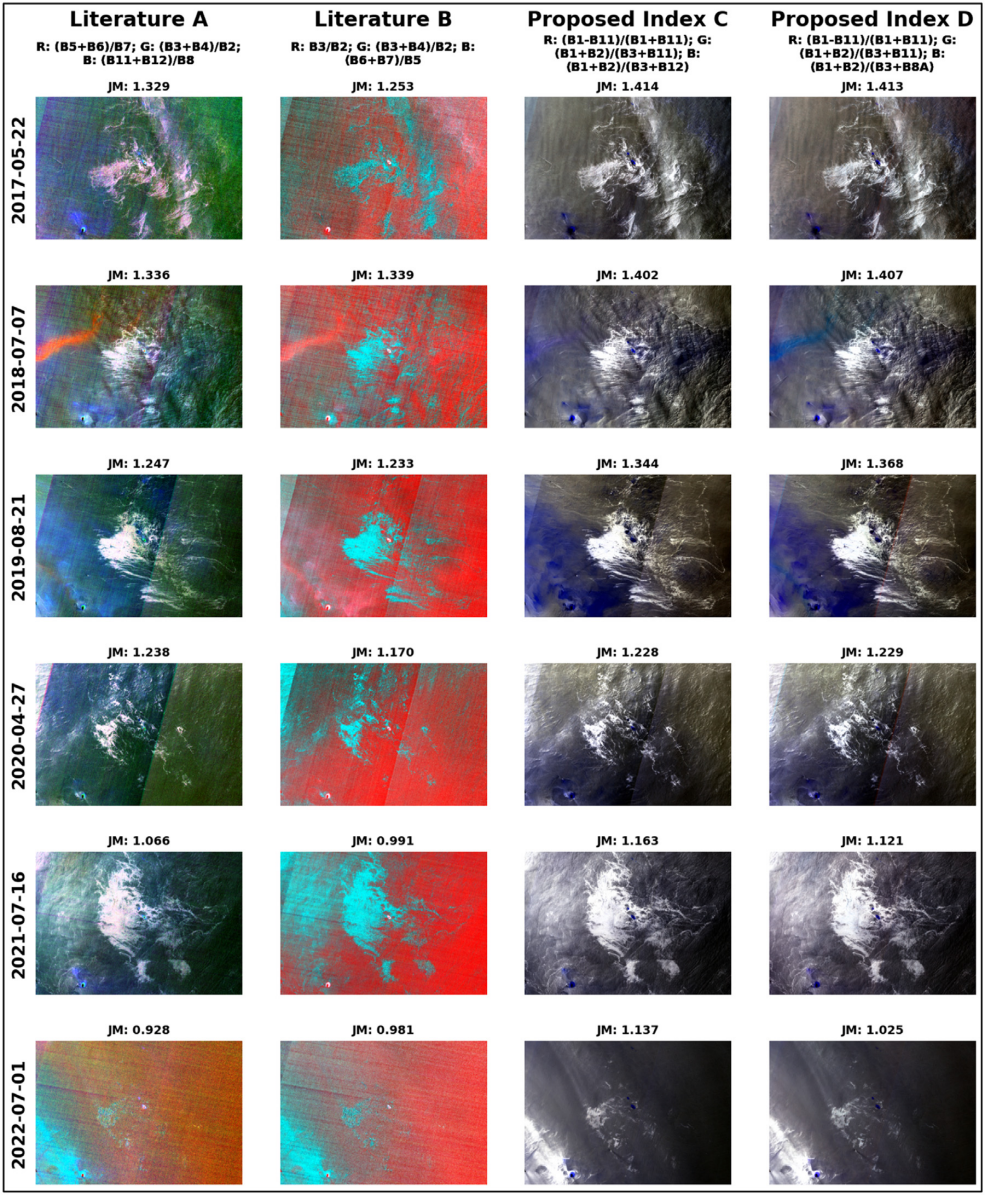
Comparative visualization of proposed 3-channel indices and previous literature indices, with corresponding JM distances.

Based on the JM distance results, the two most significant three-channel indices, as represented by Equations 8 and 7, were meticulously selected. These indices were then plotted in comparison with findings from previous studies to visually and clearly illustrate the differences that emerged, as shown in Fig. 8.

$$Index\;C\;R:\;\frac{B1-B11}{B1+B11};G:\frac{B1+B2}{B3+B11};B:\frac{B1+B2}{B3+B12}$$

$$Index\;D\;R:\;\frac{B1-B11}{B1+B11};G:\frac{B1+B2}{B3+B11};B:\frac{B1+B2}{B3+B8A}$$

It is worth noting that in the majority of test cases examined in this study, the proposed indices outperformed their counterparts from prior research. These new indices not only produced clearer and more distinguishable visual representations of the data, but they also yielded superior JM distance results. This suggests that the proposed indices are more effective in quantifying the separability between different classes of data, thereby offering a potentially significant advancement in the field.

**Fig. 9:**
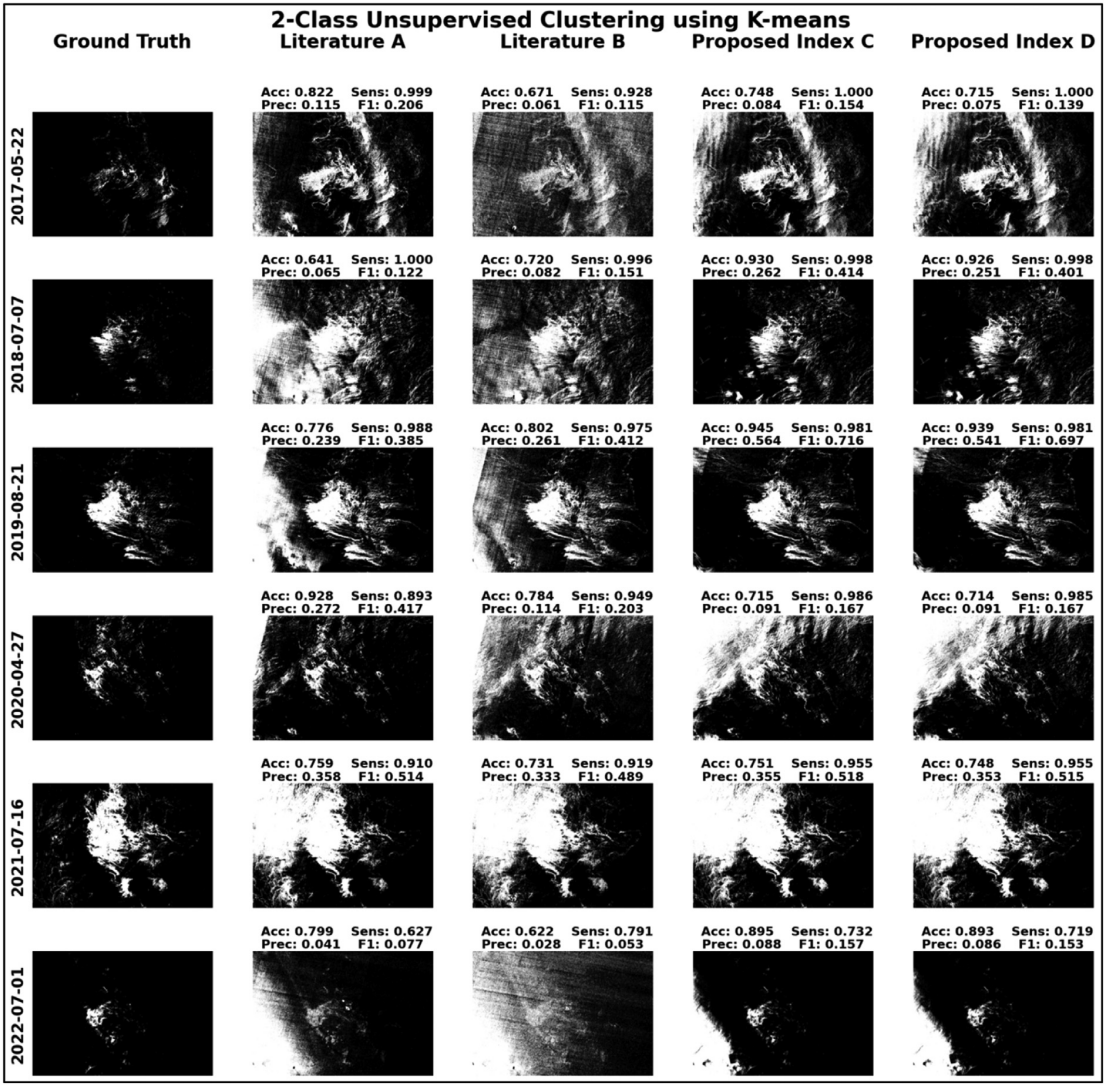
Visualization of two-class unsupervised classification executed using the k-means clustering algorithm.

**Fig. 10:**
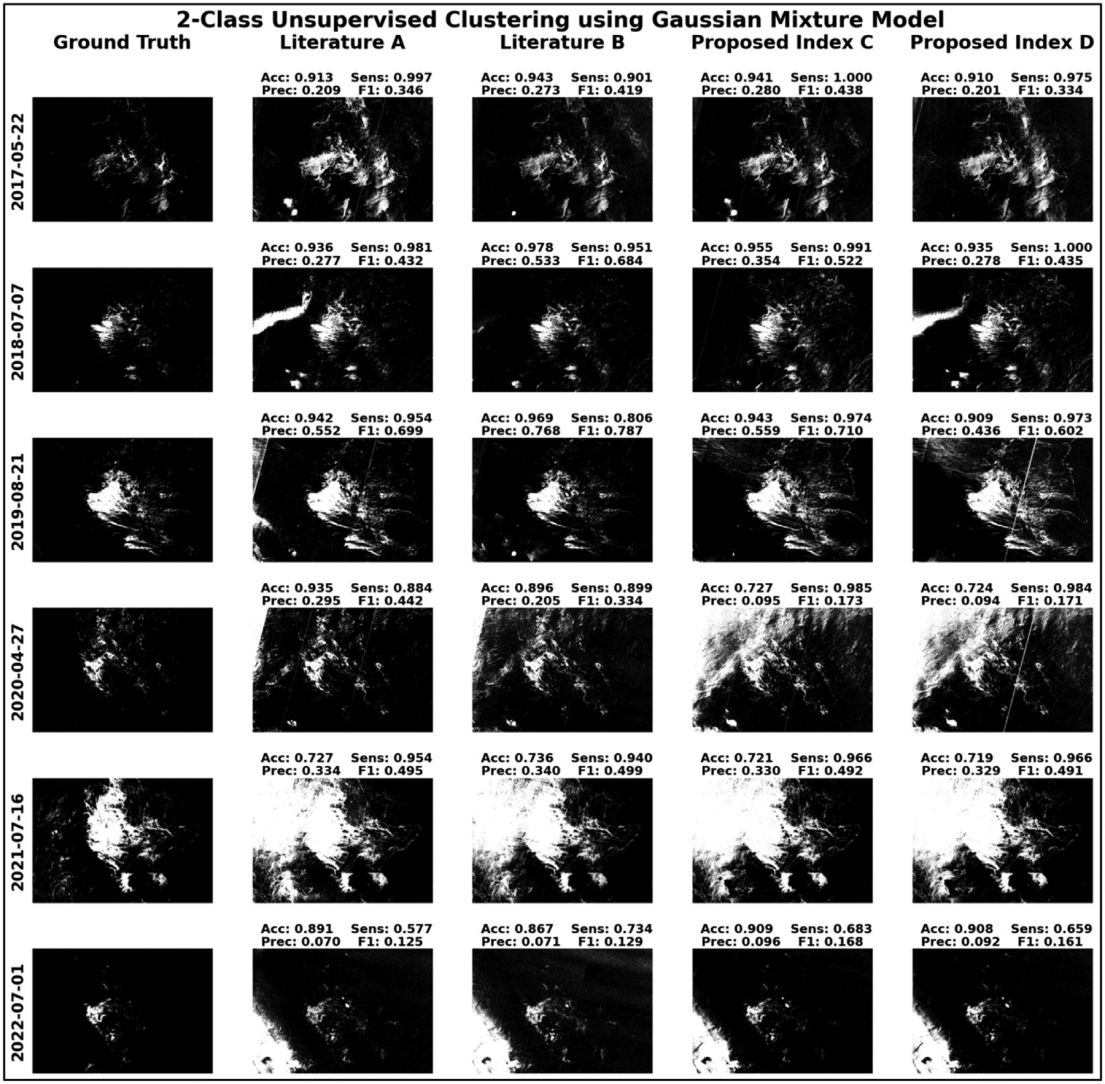
Visualization of two-class unsupervised classification executed using the Gaussian Mixture Model.

To more effectively demonstrate the efficiency of the proposed indices, Figs. 9 and 10 present the results of unsupervised two-class classification exercises, employing both k-means clustering and Gaussian Mixture Model algorithms assessed based on accuracy, sensitivity, precision, and F1 scores metrics. These figures vividly illustrate how the proposed indices enable more coherent and distinct groupings of data for most of the test cases, thereby substantiating their potential as powerful tools for enhanced data classification.

### Limitations

The presence of high-water turbidity can adversely affect the reliability of NIR bands. NIR bands are sensitive to water content, and increased turbidity limits the penetration of light into the water column. This reduction in sensitivity may compromise the detection of subtle changes associated with oil spills, potentially leading to an underestimation of the true extent of the spills. Moreover, Atmospheric conditions, including aerosols and water vapor, pose challenges to the effectiveness of SWIR bands. SWIR bands are susceptible to absorption by water vapor, and this interference can obscure the spectral signature of oil. Consequently, distinguishing oil spills from other features becomes more challenging under conditions of atmospheric interference, contributing to potential false positives or negatives in oil spill detection. The combined impact of water turbidity and atmospheric interference underscores the need for careful consideration in oil spill detection methodologies. Variations in environmental conditions may compromise the sensitivity of NIR and SWIR bands, affecting the accuracy of our proposed novel oil spill indices. Rigorous calibration and validation processes conducted under diverse environmental conditions are imperative to enhance the robustness of our methodology. Additionally, the incorporation of advanced algorithms designed to account for and mitigate the impact of environmental variations is crucial for improving the accuracy of oil spill detection in satellite imagery.

Moreover, the disparate data access policies of Landsat, Sentinel-1, and Sentinel-2 may present challenges in obtaining and synchronizing datasets. Adequate planning and coordination are crucial to ensure consistent and up-to-date data availability. Researchers should be mindful of potential gaps in the dataset, as these can impact the continuity of the analysis. Additionally, integrating data from multiple satellite platforms can be computationally intensive, necessitating robust processing capabilities. To address this challenge, researchers may consider leveraging parallel computing resources or cloud-based platforms. Implementing efficient data storage and processing strategies is essential to streamline the integration process and alleviate computational burdens.

## Conclusion and future work

The natural seepage occurring around Qaruh Island has provided a unique and invaluable context for spectral analysis of oil spills. Analyzed oil spills showed apparent distinction in reflectance from water surfaces in Sentinel-2 bands. Specifically, the oil spill samples exhibit significantly lower reflectance than the surrounding water, particularly in bands 8, 8A, 11, and 12. This distinction in reflectance offers a critical basis for differentiating between oil spills and water.

This study also sheds light on the substantial benefits of employing spectral indices to enhance data clarity. Indices allow for more precise and discernible interpretation of complex spectral data, thereby facilitating more effective and accurate oil spill detection. Despite the utility of these indices, the study highlights a notable challenge—that there is no standardized or definitive rule to guide the formulation of a new index. In response to this gap, this research endeavored to develop new indices, innovatively combining important bands identified through extensive review of previous literature.

The Jeffries-Matusita distance measure was employed to assess the separability between classes in the histograms, and interesting patterns emerged. For instance, band ratios 1 and 4 exhibited superior histogram separability compared to other tested band ratios. Furthermore, individual bands produced higher JM results than ratios 2 and 3. Notably, bands 8A, 11, and 12, when combined with bands 1, 2, and 3, yielded the highest JM values. This result aligns with the established understanding that oil spills have a pronounced effect on Near-Infrared (NIR) and Shortwave Infrared (SWIR) bands, confirming the theoretical underpinning of this study.

A particularly counterintuitive finding of this study is that incorporating a higher number of bands in the analysis did not correlate with an increase in JM values. This suggests that the selection of specific, informative bands is more critical than the sheer volume of input data. Such a revelation is highly significant for future research, as it implies that researchers can achieve effective and efficient data analysis by strategically focusing on the most informative band combinations, thereby minimizing the need for large and unwieldy datasets.

Moreover, the two-class unsupervised classification exercises conducted in this study, employing both k-means clustering and Gaussian Mixture Model algorithms, serve to effectively demonstrate the efficacy of the proposed indices. The success of these classification exercises, under two distinct algorithmic approaches, underscores the versatility and robustness of the proposed spectral indices for oil spill detection.

One of the significant avenues for enhancing the validity and reliability of the study findings is to replicate the analysis with a larger dataset. Larger datasets typically offer a broader representation, helping to iron out anomalies and provide a more generalized outcome. A critical consideration in dataset expansion is the inclusion of diverse spatial resolutions. Different satellite platforms capture imagery at varying resolutions, and a dataset that spans this spectrum enables the development of detection algorithms adaptable to multiple sensors. This approach ensures the scalability and versatility of the detection methodology. Also, Comprehensive datasets should reflect a range of temporal dynamics, encompassing different seasons, weather conditions, and time-of-day variations. This temporal diversity is essential for evaluating the robustness of detection algorithms under various circumstances and provides insights into how oil spills may manifest differently over time. Moreover, The inclusion of diverse oil types and thickness levels is paramount for a comprehensive dataset. Encompassing different oils with distinct spectral signatures and a range of thicknesses ensures the detection model's versatility across various spill scenarios. This approach accounts for the variability in visibility of spills in satellite imagery. Furthermore, diversity in environmental conditions, including water turbidity, atmospheric interference, and sunlight angles, is crucial for dataset expansion. Introducing variability in these conditions enables researchers to assess the impact of environmental factors on detection accuracy, enhancing the reliability of the model in real-world applications. Ensuring good quality of ground truth data is a fundamental consideration for dataset expansion. Meticulously annotated datasets, validated through rigorous field measurements or authoritative sources, ensure the reliability of the dataset and the precision of the detection models. High-quality ground truth data is indispensable for the accurate evaluation of detection algorithm performance. Also, considering human activities and infrastructural elements in proximity to oil spill occurrences is essential. Incorporating data from areas with diverse maritime traffic, industrial activities, and coastal infrastructure provides valuable insights into the challenges associated with detecting spills in complex environments. This context enhances the practical applicability of detection models.

While the current study primarily utilized Sentinel-2 for multispectral data, future studies can also harness the potential of Landsat imagery, given its legacy in Earth observation. Landsat, as another multispectral data provider, offers different spectral resolutions and bands that might bring in new insights when juxtaposed with Sentinel-2 data. A combined approach that integrates data from Landsat 8 with Sentinel-1 and Sentinel-2 could offer a more comprehensive temporal and spectral coverage, potentially increasing the efficiency of detection and monitoring systems. However, while the integration of data from different satellite platforms enhances the comprehensiveness of analyses, researchers must navigate challenges related to spatial, temporal, and spectral disparities. The integration of data from diverse satellite platforms, such as combining Landsat imagery with Sentinel-1 and Sentinel-2 data, introduces the challenge of varying spatial resolutions. Landsat, Sentinel-1, and Sentinel-2 satellites inherently possess different spatial resolutions. Addressing this challenge may involve the implementation of resampling techniques or the adoption of a common spatial resolution across all datasets. However, researchers should be cognizant of the potential information loss or distortion associated with these adjustments. Another critical consideration lies in the temporal frequency of data acquisition, which often differs among satellite platforms. The challenge of temporal misalignment can complicate time-series analysis and change detection efforts. To navigate this issue, synchronization methods such as interpolation or temporal alignment may be necessary. Successful integration requires careful consideration of the research objectives and the temporal characteristics inherent to each dataset to prevent misinterpretation of temporal trends. Also, The divergence in data types poses challenges in harmonizing spectral information for a coherent analysis. Techniques such as data fusion or the extraction of common spectral indices can help reconcile these spectral differences. Nevertheless, researchers must be aware of potential limitations and uncertainties introduced during this harmonization process.

Instead of using raw bands, future machine and deep learning studies could harness the selected identified band combinations as most relevant. This targeted approach might reduce noise and lead to more precise, meaningful results.

The methodology employed for developing new oil indices doesn't have to be exclusive to oil spill analysis. It can be adapted and extended to investigate other environmental and geophysical phenomena. By adjusting the parameters and leveraging relevant band combinations, current approach could provide foundational insights into a variety of studies, from vegetation health to urban development patterns.

In conclusion, this study not only furthers the understanding of the spectral characteristics of oil spills, especially in the unique context of natural seepage around Qaruh Island but also offers a set of promising tools and insights that could guide future researchers in this critical area of environmental monitoring and protection.

Ultimately, the paper advocates for collaboration with key environmental bodies such as the Egyptian Environmental Affairs Agency (EEAA) and the Saudi Environmental Society (SENS). The focus is on integrating newly developed spectral indices into existing surveillance systems, conducting on-the-ground demonstrations in vulnerable areas, implementing capacity-building programs, and advocating for policy integration. These comprehensive efforts aim to enhance environmental monitoring, improve response capabilities to oil spills, and align with broader sustainability goals, positioning the spectral indices as crucial tools for advancing environmentally conscious practices and policies.

## CRediT authorship contribution statement

**Mohamed Zakzouk:** Conceptualization, Methodology, Writing – original draft. **Islam Abou El-Magd:** Formal analysis, Supervision. **Elham M Ali:** Writing – review & editing. **Abdulaziz M Abdulaziz:** Validation, Data curation. **Amjad Rehman:** Funding acquisition. **Tanzila Saba:** Investigation, Project administration.

## Declaration of Competing Interest

The authors declare that they have no known competing financial interests or personal relationships that could have appeared to influence the work reported in this paper.

## Data Availability

Data will be made available on request.
